# Antimicrobial and Antioxidative Effects of Plant Powders in Raw and Cooked Minced Pork

**DOI:** 10.3390/foods8120661

**Published:** 2019-12-09

**Authors:** Dea Anton, Julia Koskar, Piret Raudsepp, Kadrin Meremäe, Tanel Kaart, Tõnu Püssa, Mati Roasto

**Affiliations:** 1 Chair of Food Hygiene and Veterinary Public Health, Institute of Veterinary Medicine and Animal Sciences, Estonian University of Life Sciences, Kreutzwaldi 56/3, 51006 Tartu, Estonia; raudsepp.piret@gmail.com (P.R.); kadrin.meremae@emu.ee (K.M.); tonu.pyssa@emu.ee (T.P.); mati.roasto@emu.ee (M.R.); 2 Estonian Veterinary and Food Laboratory, Kreutzwaldi 30, 51006 Tartu, Estonia; julia.koskar@student.emu.ee; 3 Chair of Animal Breeding and Biotechnology, Institute of Veterinary Medicine and Animal Sciences, Estonian University of Life Sciences, Kreutzwaldi 46, 51006 Tartu, Estonia; tanel.kaart@emu.ee

**Keywords:** blackcurrant berries and leaves, blue honeysuckle, chokeberry, rhubarb petioles and roots, tomato, polyphenols, high performance liquid chromatography–mass spectrometry (HPLC–MS)

## Abstract

It is a challenge for scientists to find new plant-based food constituents simultaneously possessing antimicrobial and antioxidative properties to prolong the shelf life of meat products. In this study, various plant powders and their blends were added to minced pork to carry out a complex study of their effect on sensory characteristics, microbial growth, and lipid oxidation of the meat in raw and cooked forms during storage. Microbiological shelf life parameters were evaluated by determining the total counts of microorganisms, yeasts, and molds. The growth potential of *Listeria monocytogenes* was estimated by challenge testing. The impact on lipid oxidation processes was assessed using thiobarbituric acid reactive substances (TBARS) and high performance liquid chromatography (HPLC) methods. The results showed that the blend of rhubarb petioles and tomato powder added a pleasant color and a combined taste to the product, similar to the taste of salt. In raw samples, considerable microbial growth inhibition was achieved with rhubarb petioles, tomato, and their mixture. Nine treatments of cooked samples had a stronger inhibitory effect on microbial growth compared to control treatments. Among all plant powders, tomato was the most effective inhibitor of yeast and mold growth. However, the challenge test revealed that *L. monocytogenes* growth in cooked samples was not inhibited during shelf life. In raw samples, rhubarb roots combined with blackcurrant or chokeberry berries effectively inhibited lipid oxidation, and in cooked samples, rhubarb petioles showed a similar effect. In conclusion, it was found that powdered plant materials are well suited for use as antimicrobial and antioxidative agents in minced meat products.

## 1. Introduction

Concerns regarding the safety and quality of processed meat products have consistently increased consumer demands for meat products enriched with natural ingredients [1,2]. 

Minced meat is particularly sensitive to microbiological spoilage and chemical changes due to the possible contamination with various microorganisms and elevated contact with oxygen. A serious problem in meat processing and storage is lipid oxidation, resulting in off-flavors, reduction in product quality, decrease in nutrient value, and increase in health risks due to the formation of toxic compounds, particularly by oxidation reactions [3]. The application of antioxidants is the best way to retard oxidation reactions and prevent the decline in product quality and shelf life. Several studies [4,5,6] have investigated the effect of the use of natural antioxidants of plant origin for such purposes. 

Nitrites are widely used for meat curing, contributing to the development of the characteristic flavor and pink color, serving as an antioxidant, inhibiting the growth of food spoilage bacteria, and most importantly, controlling the growth of *Clostridium botulinum* [7]. Sodium chloride is the most used additive in the meat industry due to its preservative and antimicrobial properties, water retention capacity, and meat flavor enhancement. However, it is also known that salt accelerates lipid oxidation [8].

The use of plant extracts with established antimicrobial and antioxidative properties can be of great significance in food preservation and for human health. Many plants are rich sources of flavonoids and phenolic and other organic acids, which act as both antioxidants and antibacterial compounds [9,10,11]. However, since most of the studies have assessed the antioxidant and antimicrobial effects of plant extracts only in vitro, the results cannot be directly applied to different food matrices. The information obtained from various studies is not always directly comparable because of the use of different methods and the significant variations in the list of antioxidants, content, and activity even between different cultivars. For example, polyphenols can possess variable antioxidant effects depending on the possibility of synergic behavior when mixed [12]. Since berries and tomatoes are consumed without peeling, we can assimilate all useful compounds that are mainly located in their skin or underneath tissues.

In the course of our in vitro experiments [13], seven plant powders were selected for further studies with meat matrices. Blackcurrant berries (*Ribes nigrum* L.) are known for their high content of vitamin C, phenolic acids, anthocyanins, and other flavonoids. Blackcurrant leaves, that have been used in sour pickled cucumber recipes since old times, contain vitamin C in lower amounts and are rich in phenolic acids, catechins, and quercetin glycosides. Garden rhubarb petioles (*Rheum rhaponticum* L.), that has the lowest total phenolic content among the studied plant parts [13], contain phenolic acids, catechins, anthocyanins, flavonols, and large amounts of organic acids, but have low vitamin C content and may contain high levels of nitrate and nitrite ions [14]. According to Raudsepp et al. [13], rhubarb roots, in contrast to petioles, have the highest total phenolic content, containing various hydroxystilbene glycosides and anthraquinones with strong and versatile antibacterial effect [15].

Chokeberry (*Aronia melanocarpa* Elliott) berries contain five-fold less vitamin C than blackcurrant berries but have a high content of β-carotene and anthocyanins, among which cyanidin glycosides most greatly contribute to the antioxidant activity of Aronia berries [16]. The edible blue honeysuckle (*Lonicera caerulea* L.) has dark purple-to-blue, oval few-seeded berries with a bitter-to-sweet taste and delicious aroma, which are rich in vitamin C, β-carotene, and various phenolics. Blue honeysuckle berries contain hydroxycinnamic acids equivalently to blackcurrants, hydroxybenzoic acids (with predominance of gallic acid), flavanes, catechins, and anthocyanins, in amounts comparable to those found in chokeberries, and smaller amounts of flavonols [12]. Tomato (*Solanum lycopersicum* L.) is a major source of lycopene and other carotenoids and a good contributor of phenolic acids and flavonoids to the human diet. Several authors [7,17,18,19,20] have reported the use of tomato paste or powder in a variety of meat products.

The present study was aimed to compare the effects of plant powders and their blends on microbial growth, lipid oxidation, and sensory parameters in raw and cooked minced pork during a defined shelf life and the effects of selected plant powders on the growth potential of *Listeria monocytogenes* in cooked pork samples.

## 2. Materials and Methods

### 2.1. Sample Preparation

Minced pork of the same lot in modified atmosphere packages (producer AS HKScan, Lääne-Virumaa, Estonia) was purchased. Fat content (27.9% ± 1.10%) and fatty acid composition (incl. linoleic acid, 2.9 ± 0.18 g/100 g) were determined at the Estonian Veterinary and Food Laboratory. The plant materials used in the study were freeze-dried using a VirTis AdVantage 2.0 EL freeze-dryer (SP Industries, Warminster, PA, USA), except for rhubarb roots and tomato, which were dried thermally at 50 °C in a dryer Binder FED 115 (Binder GmbH, Tuttlingen, Germany). The dried materials were ground using a blender (Stollar/Kinetix Control) and sieved using a laboratory sieve shaker AS300 Control (Retsch GmbH, Germany) with 2 mm mesh size. Minced meat mixtures with plant powders and other substances (Table 1) were prepared in two batches. For uniform distribution, minced meat and plant powders were thoroughly mixed with a hand mixer Clatronic HM 2935 (Clatronic International GmbH, Kempen, Germany) for 3 min at speed level 1 using kneading hooks. Minced meat without any addition was used as a control; rutin (quercetin rutinoside) and gallic acid were used as compounds representing two polyphenol classes (flavonoids and phenolic acids, respectively). Samples of 13 treatments were shaped like meatloaves (900 g each), wrapped in baking paper and aluminium foil, and cooked at 225 °C for 20 min. Internal temperature was monitored using a digital meat thermometer with a stainless-steel probe. Thereafter, the samples were cooled down to room temperature, crushed with a fork, and thoroughly mixed with the hand mixer. After each treatment, stainless bowls, forks, and mixer hooks were sprayed with ethanol and flamed to avoid contamination. The procedures were performed under the laminar-flow cabinet Kojair KR-130 (Kojair Tech Oy, Mänttä-Vilppula, Finland). All samples were divided into sterile screw-cap jars and labelled according to the storage days. The samples were stored at 4 ± 1 °C and analyzed at days 0, 2, 4, 6, and 8 (microbiological analyses of raw samples only up to the 6th day). Chemical and microbiological analyses were performed simultaneously in duplicate.

### 2.2. Sensory Evaluation

A nine-member panel (3 males, 6 females, consisting mainly of the research group members) evaluated the sensory qualities (appearance, odor, taste, overall acceptance) of the cooked meat samples on the first day right after cooking. Only the first batch was used for sensory analysis. Tasting was arranged in different sessions, comprising no more than seven samples per session to minimize “taster fatigue” [21]. Samples with rutin and gallic acid were not evaluated. Water and white bread were offered between the testings of different samples. The panel members were instructed to evaluate the aforementioned parameters in a 5-point hedonic scale (5 = excellent; 4 = good; 3 = acceptable; 2 = fair; 1 = unacceptable) [7]. Additionally, all panel members had the opportunity to leave their comments and other observations. The overall acceptability served as an indication of preference by the panel, not of consumer acceptance [19].

### 2.3. Color Measurement

The samples were packed into plastic bags, and surface color measurements were performed using an X-Rite 964 spectrophotometer (X-Rite Europe GmbH, Switzerland). The illuminant D_65_, 10° with 8° viewing angle and a 10 mm aperture was used. The values of L* (lightness), a* (redness), and b* (yellowness) were recorded (CIE Lab color system) three times in different areas of the surface of the samples on different storage days. Measurements were taken only of the first batch.

### 2.4. pH Determination

The pH values of the samples were determined in homogenates composed of 1 g of sample and 9 mL of distilled water [22]. Readings were taken with a Consort C833 digital pH-meter (Consort, Turnhout, Belgium) at room temperature, and pH-meter calibration was regularly checked.

### 2.5. Determination of the Total Phenolic Content by HPLC–MS/MS

The total phenolic contents (TPC) were determined as the areas under the HPLC UV–Vis chromatographic curves at 280 nm (AUC_280_) between 1 and 21 min using an Agilent 1100 Series liquid chromatography–tandem mass spectrometer (HPLC–MS/MS) (Agilent Technologies, Palo Alto, CA, USA) with a UV–Vis diode array detector. The results were calculated using HPLC 2D ChemStation software and expressed in arbitrary units (AU). The AUC_280_ of the meat matrix was subtracted from the respective sample AUC_280_. The chromatographic conditions are described in Chapter 2.8.

### 2.6. Microbial Enumeration

EVS-EN ISO 4833-2:2013 standard ‘Microbiology of the food chain—Horizontal method for the enumeration of microorganisms—Part 2: Colony count at 30 °C by the surface plating technique’ and EVS-ISO 21527-1:2009 standard ‘Microbiology of food and animal feeding stuffs. Horizontal method for the enumeration of yeasts and moulds—Part 1: Colony count technique in products with water activity greater than 0.95’ instructions were followed. In short, 10 g of a sample was weighed into a Stomacher bag and diluted with 90 mL of sterile, buffered peptone water (LAB204, Lab M, Lancashire, UK) to obtain the initial 10-fold dilution. Samples were blended within one minute at 230 rpm using a Stomacher™ 400 Circulator (Seward, UK). Plate Count Agar (PCA, LAB010, Lab M, Lancashire, UK) was used for the enumeration of microorganisms, and Dichloran Rose Bengal Chloramphenicol (DRBC) Agar (ISO) (LAB217, Lab M, Lancashire, UK) for the enumeration of yeast and molds. The surface plating technique was used for both enumerations by transferring 100 µL of the initial dilution and further decimal dilutions onto the surface of the agar and spreading evenly. Before incubation at the appropriate temperatures, the plates were kept for 15 min at room temperature. The PCA plates were incubated under aerobic conditions at 30 °C for 72 h, and the DRBC agar plates were incubated at 25 °C for 5 days. After incubation, the colonies were counted, and the results were expressed as decimal logarithmic units of microorganisms per gram.

### 2.7. Challenge Test

Challenge testing was performed at the Estonian Veterinary and Food Laboratory to assess the growth potential (δ) of *L.monocytogenes* in cooked minced pork samples in accordance with European Union Reference Laboratory for *Listeria monocytogenes* (EURL *Lm*) Technical Guidance Document for conducting shelf life studies on *L.monocytogenes* in ready-to-eat foods (Version 3.6, June 2014). Cooked samples (in the form of meatballs), treated with selected plant powders, were artificially contaminated using a syringe with the approximate concentration of 100 colony-forming units (cfu·g^−1^) of mixture of two *L. monocytogenes* strains (genoserotypes II (12MOB045LM) and IV (12MOB089LM)). After artificial contamination, the samples were packed into sterile screw-cap jars and kept in an incubator (Panasonic, MIR-154-PE, Hamburg, Germany) at 7 ± 1 °C. Three batches of each meat sample were analyzed at storage days 0, 7, and 14. Additionally to the numbers of *L. monocytogenes*, the total microbial count, pH, and water activity (a_w_) were determined for each batch. The growth potential of *L. monocytogenes* was determined on the base of the numbers of *L. monocytogenes*. 

### 2.8. Determination of the Lipid Oxidation Level by HPLC–MS/MS

In duplicate, 2g of sample was shaken with 4mL of methanol in a Biosan Multi RS60 (BioSan, Riga, Latvia) rotator for 30 min and centrifuged at 3200× *g* for 10 min using an Eppendorf 5810R (Eppendorf AG, Hamburg, Germany) centrifuge. The supernatant was extracted twice with 2mL of hexane to remove fat. Hexane phases were discarded, and the methanol phase was passed through a C18 SPE column (Agilent Technologies, Santa Clara, CA, USA) [23].

The main primary oxidation products of unconjugated linoleic (*all-cis*-9,12-octadecadienoic) acid (oxylipins) were identified and quantified by liquid chromatography-tandem mass spectrometry (HPLC–MS/MS) on an Agilent 1100 Series system (Agilent Technologies, Palo Alto, USA) in the negative ion mode. For the separation of the compounds, a reversed-phase column (Zorbax 300SB-C18, 2.1 × 150 mm; 5 μm; Agilent Technologies) was used in a stepwise mobile phase gradient of 0.1% formic acid and acetonitrile at the flow rate of 0.3 mL/min at 35 °C. The sample injection volume was 15 μL. For the detection and identification of substances, a 1100 Series LC/MSD Trap-XCT detector with electrospray ionization (ESI) interface was connected to the Agilent 1100 Series instrument. The total oxylipin content (TOC) was determined as the area under the extracted ion chromatogram (AUC_171_) of MS^2^ fragment ions with *m/z* = 171, characteristic for linoleic acid oxylipins formed by the 9-lipoxygenase-catalyzed pathway as well as by non-enzymatic oxidation.

### 2.9. Determination of the Lipid Oxidation Level by the Thiobarbituric Acid Reactive Substances (TBARS) Method

The concentration of secondary oxidation products of polyunsaturated fatty acids was estimated using the 2-thiobarbituric acid (TBA) method described by Modzelewska-Kapituła [22] with some modifications. To 5g of the sample, 20 mL of 4% perchloric acid and 0.25 mL of butylated hydroxytoluene (BHT) were added, and the mixture was homogenized with a Heidolph DIAX 900 (Heidolph Instruments, Schwabach, Germany) homogenizer. The homogenates were filtered through filter paper (Macherey-Nagel, Düren, Germany). Then, 5 mL of 0.02M TBA was added to 5 mL of filtrate, heated in a water bath for 60 min at 85 °C, and cooled down with tap water. The absorbance was measured at 532 nm using an AnalytikJena Specord200 spectrophotometer (AnalytikJena AG, Jena, Germany) against a blank containing 5 mL of 4% perchloric acid and 5 mL of 0.02M TBA solution. Using a standard curve prepared with 1,1,3,3,-tetramethoxypropane, the results were expressed in mg malondialdehyde (MDA) per kg of a sample.

### 2.10. Statistical Analysis

To evaluate the differences between treatments in sensory data, the generalized linear mixed model (GLMM), considering fixed effects of treatment and panelist, and random effect of session were fitted. The effects of treatment and storage time on the color measurements, total microbial counts, yeast and mold counts, and TOC and MDA were studied with GLMM considering the fixed effects of treatment and time and the random effects of triple (color measurements) or batch and duplicate (antimicrobial activity and fatty acid oxidation) variables. For all variables after modeling, the Dunnett’ post-hoc test was applied to compare the treatments with meat (control). For each treatment, also the average values (with standard errors) of pH and TPC were calculated. The relationships between the studied variables were tested with Spearman correlation analysis separately over all storage times and on the last storage day. The presence or absence of correlations allowed discussing differences between treatments and storage times. Raw and cooked meat were analyzed separately. All results were considered statistically significant at *p* ≤ 0.05. Statistical analyses were performed, and figures were constructed using the R version 3.5.3 (R Foundation for Statistical Computing, Vienna, Austria).

## 3. Results

### 3.1. Sensory Evaluation

The effect of the panelist was not statistically significant on any of the sensory traits (all *p* > 0.4), and the random session effect considered less than 10% of the overall variability of any trait.

The highest acceptability, based on appearance (color), odor, and taste, was attributed to the treatment with rhubarb(R)petioles+tomato, since tomato added a yellowish-brown color to the product, and the taste combination of tomato and rhubarb petioles imitated the taste of table salt. The treatment with 2% tomato powder had a pleasant odor and golden-orange color, but the taste was not as likeable as that of the previous combination. Eyiler and Oztan [17] reported in their study that the addition of tomato powder increased the acceptability of tested frankfurters. Hayes et al. [19] found that luncheon rolls containing 50 mg of nitrite and 1.5% tomato pulp powder had very good sensory attributes, and allowed reducing the nitrite levels by 50%.

**Figure 1:**
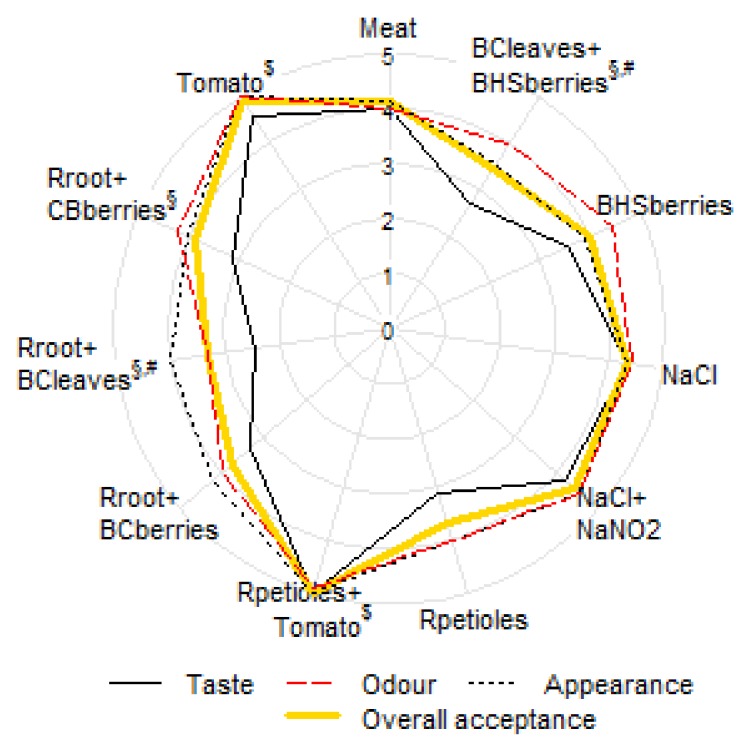
Sensory evaluation results of cooked samples; superscripts §, $, and # denote treatments with statistically significant difference from untreated meat in taste, odor, and overall acceptance, respectively. BC: blackcurrant, CB: chokeberry, BHS: blue honeysuckle, R: rhubarb, GA: gallic acid.

The highest taste scores were obtained for Rpetioles+tomato, tomato, and NaCl+NaNO_2_ treatments, and the lowest scores for Rroot+blackcurrant(BC)leaves, BCleaves+blue honeysuckle(BHS)berries, and Rpetioles (Figure 1). While some of the panelists found the cooked minced pork (control) tasteless, others even preferred that taste. The treatment with 1% of salt was generally perceived as very pleasant, but some panelists would have preferred even less salt. The color of both the control and the meat with salt was pale pink and was regarded as characteristic of minced meat. The NaCl+NaNO_2_ treatment was described as very pleasantly pink-colored, and its taste was evaluated similarly to that of the treatment with salt. In the Rroot+BCberries treatment, yellow and purple colors were mixed, which deemed pleasant to the panelists, whereas the taste was perceived as bitter-sour by some evaluators, though it was generally well acceptable. The addition of rhubarb root together with blackcurrant leaves was evaluated as sour, spicy, and herbal, characteristic of the vine leaf dolma (from the Mediterranean cuisine); the odor was characterized as herbal. The treatment with Rroot+CBberry was characterized by a pleasant taste. The addition of blackcurrant leaves together with blue honeysuckle berries was again characterized as the taste of dolma; however, for some of the panelists the taste, color, and odor were slightly unpleasant. BHSberries treatment had too sour taste and odor, and the purple color seemed too intense. Likewise, the addition of 2% rhubarb petioles tasted too sour. All berry powder treatments resulted in a purple color, which was not as well liked as the yellow-orange-golden color that was obtained with the addition of rhubarb root or tomato. The panelists rated the treatments with blackcurrant leaves as those with the least pleasant taste and odor and the lowest overall acceptance. The green color was regarded as less likeable. If plant powders will be used in production, the customers need to be trained to expect a purple or green color in meat products.

### 3.2. Color Measurements

**Figure 2:**
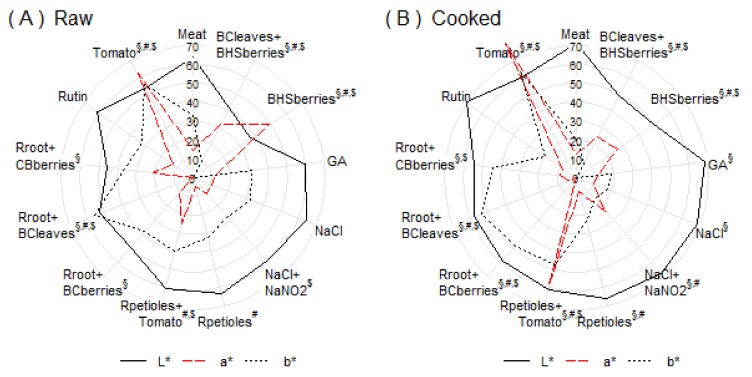
Changes in the color of the raw (**A**) and cooked (**B**) meat samples of subjected to different treatments; superscripts §, $, and # denote treatments with statistically significant difference from untreated meat in L*, a*, and b*, respectively. For better visual clarity, the redness (a*) and yellowness (b*) values were multiplied by four and two, respectively.

Consumers pay a great attention to minced meat color before they decide whether to buy a product. In our study, all added plant powders changed the color of minced meat depending on what pigments they contained (blackcurrant leaves—chlorophyll, rhubarb roots—anthraquinones, blue berries—anthocyanins, tomato—lycopene and other carotenoids). Changes in the L*, a*, b* values are shown in Figure 2. On an average, the tomato powder added the highest values to redness and yellowness and simultaneously reduced the color lightness. The same effect was reported by Deda et al. [7], Eyiler and Oztan [17], and Hayes et al. [19]. Lycopene, as a lipophilic compound, colored the fat in minced meat to a pleasant golden-orange. 

Berries of blue honeysuckle, chokeberry, and blackcurrant added redness but also reduced color lightness, giving a darker purple color to the meat. The combination of salt with nitrite slightly lowered the L* and b* values and increased the a* value, resulting in the characteristic cured-meat color. Rhubarb petioles added color lightness and yellowness and reduced redness, resulting in a pale color of the meat. Rhubarb roots, especially in combination with blackcurrant leaves, added yellowness to the meat. No statistically significant changes were observed in the color variables during the eight-day refrigerated storage period in both the raw and the cooked samples (all *p* > 0.1).

### 3.3. Changes in pH Values

**Figure 3:**
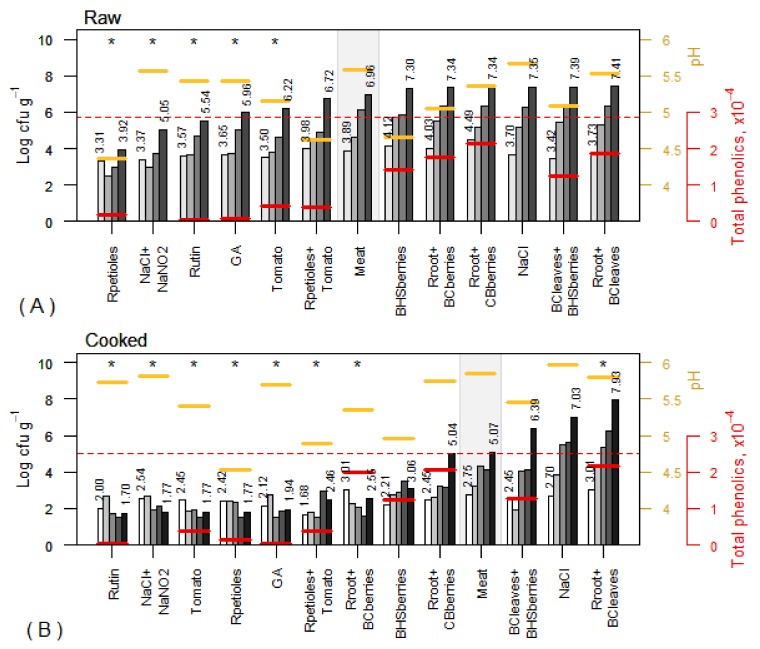
Total microbial counts (columns) of raw and cooked samples subjected to different treatments after storage times of 0, 2, 4, 6 (raw) (**A**) and 0, 2, 4, 6, 8 days (cooked) (**B**). The short horizontal lines (settled according to the secondary vertical axis) show the average pH and total phenolic contents (TPC) over storage times (standard errors for pH vary in interval 0.01–0.05, and those for TPC in the interval 0.00–0.05 × 10^4^). The treatments are ordered according to the value on the last storage day, separately for raw and cooked samples. The values on the first and the last day are presented numerically; control (meat) samples are marked with a grey background, and the stars (*) denote the treatments with a statistically significant difference with respect to control (meat) for the average level of microbial counts over storage times (*p* < 0.05, Dunnett’post-hoc test). The dotted lines denote the acceptable limits of total microbial counts on the last day of storage in the raw (5.69 log cfu·g^−1^) and cooked (5.0 log cfu·g^−1^) meat, respectively. Abbreviations are presented in Table 1.

The addition of any plant powder resulted in a decrease of the initial pH (5.65 ± 0.06) of minced meat. The results are shown in Figure 3. Rhubarb petioles caused the biggest pH drop (to 4.38 ± 0.01) when a 2% powder was added to the meat. The pH remained low (4.54 ± 0.02) in the treatment with 1% Rpetioles and 1% tomato powder. A considerable decrease in pH was also observed in the case of blue honeysuckle (0.4–0.99 units) and blackcurrant berries (0.3–0.7 units). In all cooked meats, the pH values were about 0.2 units higher. During the whole storage period, the pH of raw and cooked treated meat remained stable (standard errors 0.00–0.14), which allowed marking the pH values in the figures as short horizontal lines.

### 3.4. Total Phenolic Content

**Figure 4:**
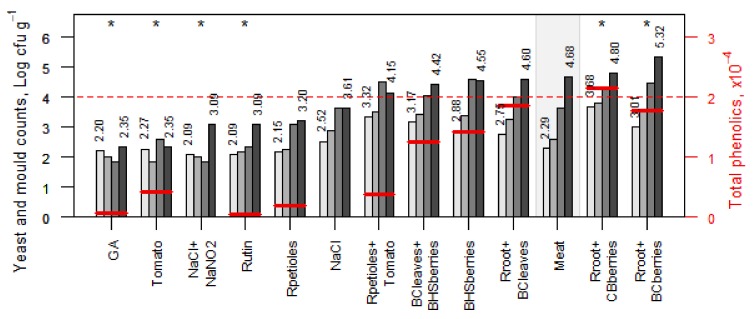
Yeast and mold counts (columns) and TPC (short horizontal lines, in arbitrary units; the standard errors for TPC vary in interval 0.00–0.05 × 10^4^) of raw minced pork samples subjected to different treatments after storage time of 0, 2, 4, 6 days. The dotted line denotes the acceptable limit of yeast and molds on the last day of storage. Treatments are ordered according to the value on the last storage day; the values on the first and the last day are presented numerically; control (meat) is marked with a grey background, and the stars (*) denote the treatments with a statistically significant difference from control for an average level of yeast and mold counts over storage times (*p* < 0.05, Dunnett’ post-hoc test). Abbreviations are presented in Table 1.

**Figure 5:**
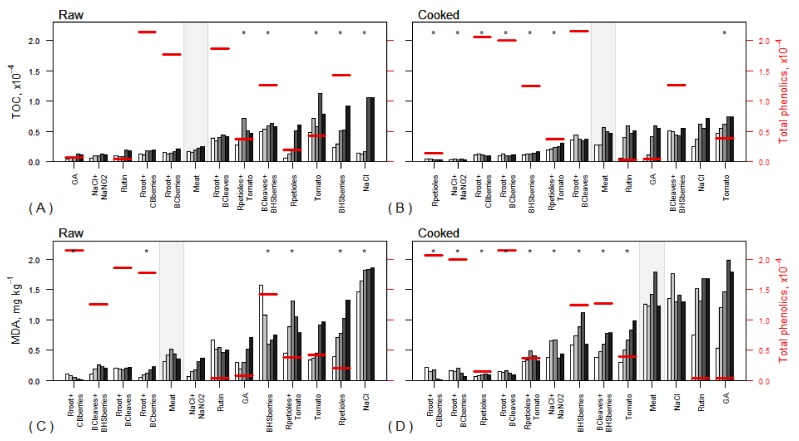
of total oxylipins (TOC) (**A** and **B**) and malondialdehyde (MDA) (**C** and **D**) in raw and cooked minced pork treated samples after storage times of 0, 2, 4, 6, 8 days. The treatments are ordered according to the values on the last storage day separately for each sub-figure; control (meat) is marked with a grey background; the stars (*) denote the treatments with statistically significant difference from control for an average levels of TOC or MDA content over storage times (*p* < 0.05, Dunnett’ post-hoc test). The short horizontal lines (settled according to the second vertical axis) show the average TPC over storage time by treatments (standard errors 0.00–0.05 × 10^4^). Abbreviations are presented in Table 1.

On an average, the highest TPC was found in the three treatments in which rhubarb root powder was combined with chokeberries, blackcurrant leaves, or berries (Figure 3, Figure 4 and Figure 5). Comparing the two treatments with blue honeysuckle berry powders, the TPC was slightly lower in the blend with blackcurrant leaves. The lowest TPC was found in the samples treated with 2% rhubarb petioles. A very small difference in TPC was observed in the sample treated with the combination of 1% rhubarb petioles+1% tomato and in that treated with 2% tomato powder. TPC was not remarkably affected by heat treatment and remained similar in raw and cooked samples.

### 3.5. Antimicrobial Activity

The total counts of microorganisms provide useful information to assess the microbiological quality of food and its shelf life. In this study, the aerobic plate count method that counts the number of mesophilic microorganisms growing under aerobic conditions was used. In general, total microbial counts increased steadily in most of the raw samples during the defined storage period of 0 to 6 days (Figure 3A). On day 0, the number of microorganisms in the tested raw samples was on average 3.31–4.49 log cfu·g^−1^ and increased to the level of 3.92–7.41 log cfu·g^−1^ by the sixth day. It is known that microbial counts in meat products may often reach 6–8 log cfu·g^−1^ [24]. However, the Commission Regulation (EC) No 2073/2005 establishes microbiological criteria for foodstuffs including process hygiene criteria for raw minced meat. Microbiological criteria have been set for aerobic colony count, with the limit of 5 × 10^6^ cfu·g^−1^ as the maximum number of microorganisms in two of five units of the samples. For the remaining three subsamples, the limit for aerobic colony count is 5 × 10^5^ cfu·g^−1^. Taking into account the official limits, it can be deduced that at the end of the defined storage period, the limits were exceeded in most raw samples, except for those treated with Rpetioles, NaCl+NaNO_2_, and Rutin (Figure 3A).

In the present study, the powder of Rpetioles appeared to be the most effective inhibitor of the growth of aerobic mesophilic microorganisms in raw minced pork (Figure 3A). The number of microorganisms increased only from 3.31 to 3.92 log cfu·g^−1^ by the sixth day. This growth inhibitory effect can be explained by the biggest drop in pH that made the environment unfavorable to microbial growth [25]. Also, this can be partially explained by the content of nitrates [14] that can be converted to nitrites in samples treated with Rpetioles. The effect of pH on microbial counts was observed only in some treatments. In spite of the low pH, BHSberries were one of the less effective bacterial growth inhibitors in raw minced pork (Figure 3A). A moderate positive correlation (r = 0.53, *p* = 0.034 in raw samples and r = 0.66, *p* = 0.005 in cooked samples) between the aerobic colony count and pH was found. 

Compared to the raw samples, larger differences in total microbial counts were observed in cooked samples (Figure 3B). On day 0, the number of microorganisms ranged between 2.0 and 3.01 log cfu·g^−1^, but depending on the sample composition, numbers varied largely from 1.70 to 7.93 log cfu·g^−1^ by the eighth day of storage. Rutin and NaCl+NaNO_2_ were the most effective microbial growth inhibitors in cooked samples. Compared to the control, seven treatments (including Tomato, Rpetioles, Rpetioles+Tomato, and Rroot+BCberries) were significantly (*p* < 0.05) more effective against microbial growth during the storage period (Figure 3B). The total number of microorganisms in all these treatments remained within the range of 1.7–2.56 log cfu·g^−1^ by the end of the storage period. It was found that compared to the control, many treatments resulted in significantly (*p* < 0.05) lower average levels of microbial counts over storage time (Figure 3A,B).

In this study, the plant materials were incorporated into minced meat in the form of a powder. It was found that especially the powdered plants were well suited for minced meat, as they are easily and uniformly mixed together. There are also other options for adding natural ingredients to meat products, such as the use of marinades with added plant materials. However, Björkroth [24] found that marinating did not increase the shelf life of meat products, because of the buffering capability of meat which neutralized the acidic marinade and resulted in the dissociation of lipophilic acids. This was the main reason why the plant powders were directly mixed with minced meat in the present study. 

The changes in yeast and mold counts of raw samples during refrigerated storage are shown in Figure 4. The initial counts of yeasts and molds were between 2.09 and 3.68 log cfu·g^−1^. The high initial yeast and mold counts were caused by natural contamination of the plant powders, as no sterilization of the plant materials was applied. In a majority of samples, the initial counts increased steadily by the sixth day of storage, with the exception of samples treated with GA and Tomato. Therefore, a control measure, e.g., irradiation, should be applied, which eliminates or significantly reduces microbial contamination without affecting the beneficial properties of plant powders. No official criteria for yeast and mold counts in raw minced meat have been established, but the 4.0 log cfu·g^−1^ limit at the end-point of shelf life was set in the present study. The results showed that by the last day of storage, this limit was exceeded in seven treatments (Figure 4). Interestingly, a slight decrease in yeast and mold counts was observed in samples treated with tomato (from 2.27 to 1.85 log cfu·g^−1^ on the second day), NaCl+NaNO_2_ (from 2.09 to 1.85 log cfu·g^−1^ on the fourth day), and gallic acid (from 2.20 to 1.85 log cfu·g^−1^ on the fourth day). Also, these treatments showed a strong antimicrobial effect against aerobic mesophilic microorganisms. The highest yeast and mold growth inhibition was detected in samples treated with GA, Tomato, NaCl+NaNO_2_, Rutin, Rpetioles, and NaCl, in which the set limit was not exceeded at the end of storage. 

In the cooked samples, the yeast and mold counts mostly remained below the limit of detection (100 cfu·mL^−1^). Only in the samples containing NaCl and NaCl+NaNO_2_, the total yeast and mold counts ranged from 100 to 200 cfu·g^−1^ during the eight-day storage period. Very low yeast and mold counts can be explained by the efficient thermal processing that eliminated most of the yeasts and molds in cooked samples, which, in turn, facilitated the growth of aerobic mesophilic microorganisms.

In general, in many samples, plant powders reduced the mesophilic microorganism counts as well as yeast and mold counts compared to the control during a defined storage period, which suggest different inhibition mechanisms. The findings showed that the effect of polyphenols was modest. Petioles, which had the lowest total polyphenols levels, showed the strongest inhibition ability of microbial growth in both raw and cooked samples.

Yeast and mold growth was properly inhibited in samples containing pure chemical compounds (GA, NaCl, NaCl+NaNO_2_, Rutin). Despite its low polyphenol content, the inhibition effect was remarkable in tomato-treated samples. In contrast, the samples containing berry powders demonstrated relatively high counts of mesophilic microbes, yeasts, and molds. In the case of raw samples, this is probably related to the high initial contamination of the plant materials (Figure 4). In addition, even if the meat samples enriched with berry powders contained the highest levels of polyphenols, little microbial growth inhibition was observed. This contradiction can be explained by a nutritive effect of the powders for different microorganisms. The latter also explains the positive correlation between TPC and the total yeast and mold count (r = 0.68, *p* = 0.004), as well as between TPC and the total microbial count in raw (r = 0.69, *p* = 0.003) and cooked (r = 0.75, *p* < 0.001) samples.

In this study, BCleaves had no inhibitory effect on microbial growth. Our previous in vitro study [13] found that petioles and the roots of garden rhubarb had a high antibacterial and antioxidative activity and suggested them as good candidates to use in meat products. Obviously, not always the promising results of in vitro studies are repeatable in food matrices. Rhubarb roots, that had a strong in vitro antimicrobial effect, showed a moderate or even weak effect in the present study. This can partially be explained by the findings of Gyawali and Ibrahim [26], who showed that in food matrices, many bioactive compounds are bound to the hydrophobic moieties of proteins and lipids, which restricts the availability of these natural antimicrobials.

In addition to the antimicrobial and antioxidant effects, the sensory properties of treated foods should also be taken into account. Frequently, high concentrations of certain plant powders are organoleptically unacceptable in meat products. However, our results are in a good agreement with Tiwari et al. [27], who found that in optimal concentrations, natural antimicrobials can inactivate microorganisms, without impairing the organoleptic properties of food.

### 3.6. Challenge Test

The results of the challenge test (Table 2) showed that the growth potential of *L. monocytogenes* was high in all cooked meat samples. It can be concluded that plant powders alone are incapable of inhibiting the growth of *L. monocytogenes* in meat products prepared without food additives, e.g., preservatives. The growth potential values obtained for *L. monocytogenes* indicate that post-heat treatment contamination of meat products enriched with plant powders must be avoided. To ensure microbiological food safety of similar ready-to-eat meat products that support the growth of *L.monocytogenes*, the absence of the pathogen in 25 g of sample is required before the food has left the immediate control of the food producer. Relatively short shelf life should be applied additionally to the legal limit (not detected in 25 g).

The Rpetioles+Tomato powder did not stop the growth of *L. monocytogenes* but had the strongest inhibitory effect on the growth of this bacterium compared to other samples. Generally, low total microbial counts were determined throughout the storage period in the challenge test, but this could be due to the high concentration of *L. monocytogenes* and the related competition effect within the microbiota. It was found that cooked minced pork enriched with selected plant powders can support the growth of *L. monocytogenes*. The calculation of the maximum growth rates, based on the values obtained in the challenge test, revealed that cooked meat samples enriched with selected plant powders were able to support the growth of *L. monocytogenes*. Therefore, “zero tolerance” for *L. monocytogenes* in such meat products shall be established, in accordance with the food safety criteria laid down in the Commission Regulation (EC) No 2073/2005 on microbiological criteria for foodstuffs.

### 3.7. Fatty Acid Oxidation

The free polyunsaturated fatty acids (PUFA), especially linoleic acid (LA), are prone to (per)oxidation by chemical, enzymatic, and combined routes [4,28]. During oxidation, various toxic compounds, particularly leukotoxin diols and aldehydes, are formed that substantially reduce the quality and safety of meat products. Oxidation starts with the formation of acid hydroperoxides and continues in two main phases which comprise a number of steps, different enzymes, and free radicals. Throughout the first phase, the hydrocarbon backbone of LA remains intact, and oxygen atom(s) are linked by double bond(s) to sequentially form a set of various oxylipins.

During the second phase, aldehydes and other toxic foul-smelling breakdown products of oxylipins are formed [29]. The thiobarbituric acid reactive substances (TBARS) test is generally used to determine the content of MDA, one of the principal secondary products of lipid oxidation.

Due to the different mechanisms of their antioxidant effects, the plant powders used in the present work behaved differently in the raw (Figure 5A,C) and cooked (Figure 5B,D) samples. While various enzymatic and chemical mechanisms are usually competing with each other in raw meats, the enzymatic routes of PUFAs oxidation are excluded in cooked samples. In the first case, inhibitors of oxidation enzymes (lipoxygenases, cyclooxygenases, CYP450), as well as free-radical scavengers, ferric ion sequesterers, etc., have their role in the extremely complicated antioxidant effect of plant mixtures. The values of the TOC, MDA, and TPC in the raw and cooked samples are presented in Figure 5.

In the raw samples, where enzymatic and chemical mechanisms are intertwined, GA, NaCl+NaNO_2_, Rutin, Rroot+CBberries, and Rroot+BCberries appeared to be the most effective inhibitors that guaranteed lower TOC levels compared to the control throughout the entire storage period, (Figure 5A). The last two blends contained potent free-radical scavengers (anthocyanins) from the berries [30], as well as a number of antioxidants (polyphenols) from rhubarb root, like hydroxystilbenes, piceatannol glucoside, and galloglycosides of piceatannol, resveratrol, rhapontigenin, and deoxyrhapontigenin, as well as resveratrol dimers [13,15,31]. Flavonoids are known to inhibit oxidation chain initiation and prevent chain propagation through metal chelation or free-radical scavenging mechanisms, whereas phenolic acids act as antioxidants via free-radical trapping mechanisms [32]. The actual mechanism may depend on the concentration of the antioxidant and the type of free radical. The structural feature, responsible for the antioxidative and free-radical scavenging activity of gallic acid consists of three adjacent hydroxyl groups. The antioxidant activity of gallic acid is based on a single-electron transfer mechanism [33]. 

In the raw samples, higher TOC values compared with the control were associated with seven treatments. This promoting effect can be linked with the higher acidity of these mixtures compared to other plant powders used in the tests, which may promote oxidation of PUFAs [4]. The higher TOC values in the samples treated with BCleaves, tomato, and BHSberries can also be explained by LA oxylipins originating from blackcurrant leaves or tomato seeds [34] and cuticles, rich in unsaturated fatty acids, particularly linoleic acid. Compared to chokeberry and blackcurrant berries, blue honeysuckle berries do not contain delphinidin [13], which possesses a slightly higher antioxidant activity than cyanidin, owing to the presence of vicinal OH groups [35]. Altogether, tomato, rhubarb petioles, and blue honeysuckle berries exerted a pro-oxidative effect in meat mixtures (high TOC and MDA values, Figure 5A,C). However, the highest TOC values were registered when NaCl was added to minced pork. Mariutti and Bragagnolo [8] have reported studies where NaCl acted as a pro-oxidant agent in meat and meat products, probably because of its capacity to disrupt cell membrane integrity, liberate iron ions from iron-containing molecules, and/or inhibit the activity of antioxidant enzymes. 

At the same time, the BCleaves+BHSberries treatment was associated with a several-times lower MDA value than the treatment with 2% BHSberries (Figure 5C). This can be explained by the presence of powerful in vitro antioxidants, e.g., catechin gallate, chlorogenic acid, myricetin, and quercetin glycosides in blackcurrant leaves [13,36] that help slow down the oxidation processes in the second phase of oxidation. This can also be explained by the influence of environmental acidity on the predominant structural forms of anthocyanins present in the mixture [37]. At pH 5, anthocyanin molecules are predominantly in the form of pseudobases, lacking a conjugation system over the molecule, which is necessary for the formation of a stable secondary radical after scavenging very reactive oxygen radicals. However, at pH6, anthocyanins are preferably in the form of chalcones, characterized by the presence of continuous resonance structures involving all nine double bonds. A strong negative correlation (r = −0.74, *p* = 0.001) between pH and MDA content was established.

Concerning the second phase of linoleic acid oxidation in both raw and cooked samples, the lowest MDA levels (Figure 5C,D) were obtained for the treatment with Rroot+CBberries, the only treatment characterized by decreasing MDA values during the storage period. This phase of oxidation was effectively inhibited by the three treatments containing rhubarb root, and the treatment with BCleave+BHSberries (Figure 5C). While the treatment with Rroot+BCleaves kept the oxidation levels stable throughout the storage period, the treatment with Rroot+BCberries efficiently inhibited the fatty acid oxidation rate on the first day but did not prevent its slow increase during the following storage days. Similar oxidation levels and an upward trend were observed in samples treated with NaCl+NaNO_2_. Rutin kept the MDA levels slightly higher with respect to the control but stable during the whole storage period. Gallic acid appeared to be efficient on the first days, but after the fourth day, the MDA content began to increase. Tomato and Rpetioles, either separately or in a blend, promoted oxidation. BHSberries, when 2% of powder was incorporated in the minced meat, promoted lipid oxidation, but BCleaves+BHSberries effectively inhibited MDA formation. Although NaCl+NaNO_2_ retarded the oxidation process, the highest lipid oxidation level was registered in samples treated with NaCl (Figure 5C). An overall strong negative correlation was observed between the TPC and PUFA (per)oxidation markers: the total oxylipins content (r = −0.69, *p* = 0.003) and MDA content (r = −0.90, *p* < 0.001) that characterize the main oxidation phases in raw samples. 

In the thermally processed samples (Figure 5B,D), where only chemical oxidation occurred, Rpetioles, and Rhubarb root blended with chokeberry berry, blackcurrant berry, or blackcurrant leaves were among the strongest inhibitors of the formation of primary and secondary oxidation products of LA. Only in some treatments, the TOC and MDA values were higher than in the control. Comparing the effectiveness of Rpetioles, Rpetioles+tomato, and tomato, the TOC and MDA values were the highest in samples treated with tomato powder alone, reduced by a half when Rpetioles were added to the tomato powder, and the lowest in the treatment with Rpetioles alone. Like in the raw samples, significant lipid oxidation processes occurred in the meat containing salt. Surprisingly, the antioxidative effect of rutin and gallic acid was not as strong in cooked samples as in the raw ones, although their concentration did not decrease substantially during cooking. 

### 3.8. Antimicrobial and Antioxidant Effect

**Figure 6:**
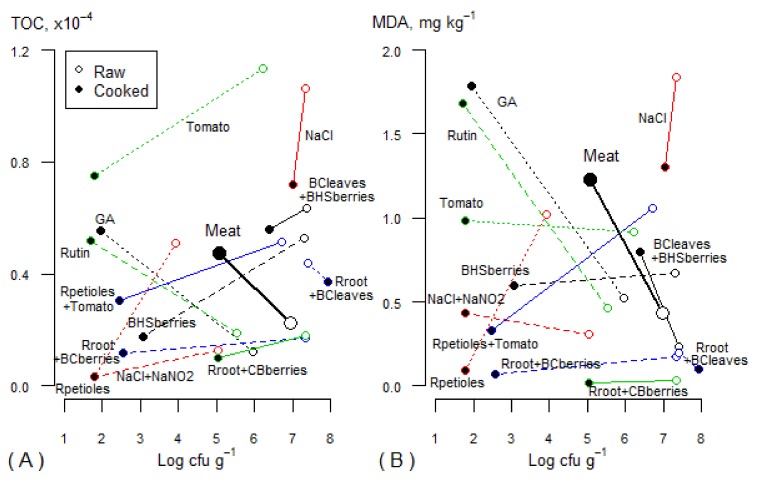
Association between the content of (**A**) TOC and (**B**) MDA and total microbial counts (Log cfu·g^−1^) in raw and cooked minced pork on the last storage day, depending on the treatment. Each dot marks the average values of raw and cooked minced pork samples corresponding to the same treatment and is joined with a line (shorter lines indicate higher similarity between raw and cooked minced pork samples for that treatment). Control (meat) samples are denoted with bigger symbols; for other treatments, different line styles and colors are used to facilitate their distinction (same line styles and colors are used for the same treatments in Figure 6A,B). Abbreviations are presented in Table 1.

The plant powders acted differently in raw and cooked samples and, in several cases, also during the first and second phase of lipid oxidation (Figure 6). Several cooked treatments, represented by the black dots locating in the left corner of Figure 6A, had lower TOC values and total microbial counts compared with the control (meat). The treatment with NaCl strongly differentiates from the others, showing the highest TOC and MDA values in both raw and cooked samples. Also, the inhibition of microbial growth was very weak. However, NaNO_2_ and NaCl together exhibited a strong antimicrobial effect. Both in raw and cooked treatments, incorporation of BCleaves resulted in high microbial loads, but the oxidation process was inhibited when BCleaves were mixed with Rroot. Furthermore, when BCleaves were mixed with BHSberries, a weak inhibitory effect on microbial growth was observed. BHSberries alone (2%) had a much stronger antimicrobial effect in the cooked samples, and the MDA content remained stable in both raw and cooked samples. 

Gallic acid and rutin behaved almost identically. Both had a higher antimicrobial effect and led to slightly lower TOC values compared with control but, in the cooked samples, they strongly promoted the formation of MDA in the second phase of the oxidation process (Figure 6B). Rroot+CBberries showed to be highly effective oxidation inhibitors, keeping TOC and MDA level low. However, the inhibitory effect on microbial growth of this combination was relatively weak. Rpetioles+Tomato, Tomato, and Rpetioles showed a similar inhibitory effect in the cooked samples, whereas Rpetioles alone had the highest inhibitory effect on microbial growth in the raw samples. The TOC values for Tomato (2%) were twice as high compared to those for Rpetioles+Tomato (1% + 1%), due to the increased amount of tomato powder incorporated (Figure 6A). Rpetioles alone (2%) showed a high inhibitory effect on microbial growth in both raw and cooked samples and was among the most effective inhibitors of the oxidation processes in the cooked samples.

## 4. Conclusions

It was rather complicated to find plant mixtures that simultaneously suppress microbial growth and lipid oxidation in the meat products studied. Plant powders used in this work showed different behaviors in raw and cooked samples and during the different phases of lipid oxidation.

Rpetioles showed an inhibitory effect comparable with that of NaCl+NaNO_2_ in raw samples. Tomato, Rpetioles, and their combination, as well as Rroot+BCberries and BHSberries had a similar microbial effect in cooked samples. Tomato was found to most effectively inhibit the growth of yeasts and molds, having an antifungal effect similar to that of NaCl+NaNO_2_. The challenge test revealed no effect on the growth potential of *L. monocytogenes*. Nevertheless, in six treatments out of eight, the addition of plant powders to meat helped prolong the shelf life of the products by keeping the total microbial counts low. The addition of a small amount of salt resulted even in a shorter shelf life from both the microbiological and the chemical point of view.

The strongest antioxidant effect was attained with chokeberry or blackcurrant berries in combination with rhubarb root.

It must be highlighted that the combination of rhubarb petioles with tomato provided an attractive color to meat/fat and a pleasant taste even without added salt. This combination of plant powders is promising for developing meat products of improved quality and with reduced salt content.

## Figures and Tables

**Table 1:** Plant powders and other substances used in minced meat treatments. BC: blackcurrant, CB: chokeberry, BHS: blue honeysuckle, R: rhubarb, GA: gallic acid.

No	Abbreviation	Composition
1	Meat (Control)	Minced meat
2	NaCl	Minced meat with 1% sodium chloride
3	NaCl+NaNO_2_	Minced meat with 1% sodium chloride and 150 mg/kg sodium nitrite
4	Rroot+BCberries	Minced meat with 1% rhubarb root+1% blackcurrant berries
5	Rroot+BCleaves	Minced meat with 1% rhubarb root+1% blackcurrant leaves
6	Rroot+CBberries	Minced meat with 1% rhubarb root+1% chokeberry berries
7	BCleaves+BHSberries	Minced meat with 1% blackcurrant leaves+1% blue honeysuckle berries
8	Rpetioles+Tomato	Minced meat with 1% rhubarb petioles+1% tomato
9	BHSberries	Minced meat with 2% blue honeysuckle berries
10	Rpetioles	Minced meat with 2% rhubarb petioles
11	Tomato	Minced meat with 2% tomato
12	Rutin	Minced meat with 80 mg/kg rutin
13	GA	Minced meat with 80 mg/kg gallic acid

**Table 2:** Results of the challenge test.

Sample	Storage Day	pH *	Water Activity * a_w_	Total Count (cfu·g^−1^)	Growth Potential δ (log cfu·g^−1^) **
Meat	0	6.19 ± 0.021	0.986 ± 0.003	2	5.72
	7	6.19 ± 0.021	0.986	2.2 × 10²	
	14	6.19 ± 0.007	0.982 ± 0.001	9.4 × 10³	
NaCl+NaNO_2_	0	6.17	0.972 ± 0.001	<1	5.97
	7	6.22 ± 0.014	0.976 ± 0.001	7.5 × 10¹	
	14	6.18 ± 0.007	0.976	<1	
Rpetioles+Tomato	0	5.58 ± 0.021	0.985	9	4.54
	7	5.50 ± 0.014	0.984	2	
	14	5.37	0.982 ± 0.001	4	
Rroot+BCberries	0	5.66 ± 0.014	0.984	6.1 × 10²	5.30
	7	5.68 ± 0.007	0.983 ± 0.001	4.5 × 10¹	
	14	5.71	0.981	7.1 × 10¹	
Tomato	0	5.90 ± 0.007	0.983	3	5.67
	7	5.84 ± 0.007	0.982	2	
	14	5.85 ± 0.007	0.978 ± 0.001	1	

* Values are mean ± SD (standard deviation) obtained from analyses performed in duplicate. Zero SD values are not shown. ** When δ > 0.5 log cfu·g^−1^, the food is classified as “ready-to-eat food able to support the growth of *L. monocytogenes*”.
